# *In vivo* Illustration of Altered Dopaminergic and GABAergic Systems in Early Parkinson's Disease

**DOI:** 10.3389/fneur.2022.880407

**Published:** 2022-05-17

**Authors:** Hirotsugu Takashima, Tatsuhiro Terada, Tomoyasu Bunai, Takashi Matsudaira, Tomokazu Obi, Yasuomi Ouchi

**Affiliations:** ^1^Department of Neurology, Shizuoka Institute of Epilepsy and Neurological Disorders, Shizuoka, Japan; ^2^Department of Biofunctional Imaging, Preeminent Medical Photonics Education and Research Center, Hamamatsu University School of Medicine, Hamamatsu, Japan; ^3^Hamamatsu PET Imaging Center, Hamamatsu Medical Photonics Foundation, Hamamatsu, Japan

**Keywords:** Parkinson's disease (PD), flumazenil, CFT, frontal assessment battery (FAB), GABA, positron emission tomography

## Abstract

**Background:**

Changes in γ-aminobutyric acid (GABA) function are noted in patients with Parkinson's disease (PD) who have some non-motor impairments. However, dopamine-related GABA function and GABA-related cognitive changes are still unclear.

**Methods:**

Thirteen drug-naive early-stage PD patients underwent a series of PET scans with [^11^C]flumazenil(FMZ) and [^11^C]CFT. The [^11^C]FMZ binding potential (BP_ND_) derived from a Logan plot analysis was compared between PD patients and age-matched controls. The [^11^C]CFT radioactivity relative to the cerebellar counterpart was estimated as a semiquantitative value [^11^C]CFT SUVR. Correlations between [^11^C]FMZ BP_ND_ and [^11^C]CFT SUVR in the same region of interest were also examined.

**Results:**

In patients in the PD group, [^11^C]CFT SUVR was significantly lower in the putamen. The levels of [^11^C]FMZ BP_ND_ in the cerebral cortex (frontal lobe dominancy) and the affected-side putamen were also reduced. In addition, [^11^C]CFT SUVR was negatively correlated with the [^11^C]FMZ BP_ND_ level in the affected-side putamen. In patients in the PD group, the total frontal assessment battery (FAB) score was positively correlated with the [^11^C]FMZ BP_ND_ in the frontal region.

**Conclusion:**

GABAergic dysfunction coexists with dopaminergic loss not only in the putamen but also over the extrastriatal region in patients with early PD and is related to frontal dysfunction. The negative correlation of [^11^C]CFT SUVR with [^11^C]FMZ BP_ND_ in the affected putamen suggests that a greater dopaminergic demise would decelerate GABA release (or an increase in tracer binding), resulting in persistent failure of the GABAergic system in PD patients.

## Introduction

Parkinson's disease (PD) is a neurodegenerative disorder that is caused by the loss of dopaminergic neurons in the substantia nigra pars compacta. Striatal dopamine depletion is thought to lead to cardinal motor symptoms of PD, including tremor, rigidity, bradykinesia and postural instability. Although the key symptoms of PD are motor deficits, patients with PD frequently show various degrees of non-motor symptoms (1), such as rapid eye movement sleep behavior disorder, depression, reduced sense of smell and constipation; these symptoms often precede the onset of motor impairment. While dementia in patients with PD is thought to occur in the late stage of the disease course, it has been reported that altered frontal functions deduced from poor performance in working memory tasks occur in patients with early-stage PD (2). Some cognitive impairments in patients with early PD are ascribed to the loss of dopaminergic neurons because dopaminergic medication partially improves some cognitive decline. However, it was reported that dopaminergic medication in patients with PD has no effect on recognition memory or cognitive flexibility and worsens impulsivity (3). A loss of such cognitive resiliency of the brain would insinuate improper function of the inhibitory neuronal function governed chiefly by the γ-aminobutyric acidergic (GABAergic) system.

GABA is the primary inhibitory neurotransmitter in the human brain. Previous studies on PD with magnetic resonance spectroscopy (MRS) and postmortem brain samples showed that GABA concentration increased in the basal ganglia in PD patients (4, 5) and that a reduction in the motor cortex GABA level was associated with motor severity (6). In an animal model, L-DOPA-induced dyskinesia is associated with excessive GABA release in the medial globus pallidus (7), and GABA decline is suggested to accelerate disease progression (8). In general, GABAergic dysregulation is likely associated with cognitive impairment in neurodegenerative disorders. For example, it has been reported that a decrease in GABA in the inferior frontal gyrus is associated with impulsivity due to frontotemporal lobar degeneration (9) and that GABA is reduced in the parietal region in patients with Alzheimer's disease (10). In PD, a reduced GABA level in the visual cortex is associated with hallucinations (11), and genes associated with GABA transmission are downregulated in the frontal cortex in DLB (12). As described, the GABAergic system is deeply involved in the control of cognitive function. However, questions remain about the interaction of the striatal dopaminergic system with the GABAergic system and about the weight of these two systems on the frontal cognitive decline seen in early PD.

One classical model of interpretation of dopamine and GABA interactions shows that striatal dopamine depletion is thought to cause the activation of indirect pathways, resulting in increased GABA in the striatum (13) and hence inhibiting the thalamus and the motor cortex, which leads to the suppression of movement. While GABA control was once thought to reside downstream of the dopaminergic system, a recent study showed that pharmacological stimulation of the striatal GABA-A receptor inhibits dopamine release in the striatum and dopamine neurons coreleased dopamine and GABA in an animal experimental setting (14). GABA and dopamine systems are suggested to interact intricately with each other. In the clinical setting, however, studies focusing only on GABA thus far cannot explore an interaction between dopamine and GABA in the living brain of PD patients. Since it is expected that a prolongation of an inhibitory drive on the cortex via the basal ganglia-cortical circuit would lead to dysfunction of the frontal cortex, a close look simultaneously at the GABAergic systems present in the cortical interneurons and in the basal ganglia will be important in view of dopamine demise in PD to help understand the pathophysiology of PD showing cognitive decline at an early stage of the disease.

The purpose of our study was to explore the *in vivo* changes in the GABAergic and dopaminergic systems. Unlike studies with MRS to estimate the GABAergic change, *in vivo* investigation of GABA-A receptor can estimate the GABAergic function indirectly. Since there is no specific tracer that depicts the GABAergic system, we chose to perform positron emission tomography (PET) with [^11^C]flumazenil(FMZ) that binds to the benzodiazepine binding site on the GABA-A receptor and [^11^C]CFT that binds to dopamine transporter, respectively, in early-stage PD patients. We also aimed to clarify their relationships with cognitive impairment to determine the contributions of these systems to cognitophysiology in early PD.

## Materials and Methods

### Participants

We studied 13 drug-naive PD patients (5 males, 8 females, 62.1 yrs ± 7.2 SD) and 15 healthy control subjects (6 males, 9 females, 55.8 ± 11.5). All PD patients and six healthy control subjects out of 15 people completed the double-tracer measurement in this study. The remaining 9 control subjects underwent the [^11^C]FMZ PET scan only.

We obtained written informed consent for the present study, which was also approved by the ethics committee of Hamamatsu Medical Center. Clinical assessments of each PD patient were performed with the Unified Parkinson's Disease Rating Scale (UPDRS). Patients with limb tremors, rigidity, bradykinesia and confirmed by a tentative trial of L-dopa for clinical diagnosis of PD were recruited from our main hospital or the neighboring clinics, and healthy controls free from any neurological complications were recruited by in-house advertisement. All participants underwent magnetic resonance imaging (MRI), a neuropsychiatric examination, and a blood test to exclude the possibility of any accompanying diseases. A research neurologist or psychiatrist administered the Depression Module of the Structured Clinical Interview for DSM–IV (SCID) to exclude the possibility of psychiatric disorders. Cognitive and behavioral functions were evaluated by the Mini Mental State Examination (MMSE) to exclude dementia and Frontal Assessment Battery (FAB) to evaluate exective function. Unfortunately, few patients were examined with Montreal Cognitive Assessment (MoCA) that can estimate the level of mild cognitive impairment. The neuropsychological assessment results for all PD patients are shown in Table 1. Neuropsychological evaluation was performed just before the PET examination. The UPDRS scores varied from 11 to 32, and the UPDRS part-III scores varied from 5 to 23. The duration of disease was from 3 to 36 months (mean: 13.8 months). The disease duration was defined by the time from initial symptoms. All PD patients were diagnosed as having a clinical severity of Hoehn-Yahr stage (H&Y) 1 or 2.

**Table 1:** Demographic and clinical characteristics of participants in the Parkinson's disease (PD) group and control group.

	**PD group**	**Control group**	**Normal range**	***P*-value**
Age (years)	62.1 ± 6.9	55.8 ± 11.5		n.s.
Men/women (number)	5/8	6/9		n.s.
Disease duration (months)	13.8 ± 8.1			
Hoehn and Yahr (I/II)	8/5			
Unified PD rating scale part III	12.2 ± 4.9			
Mini-mental state examination (/30)	27.4 ± 3.5		>23	
Frontal assessment battery (/18)	13.9 ± 3.5		15.3 ± 1.2^#^	0.006^*^

*Data are presented as the mean ± SD (range)*.

# *Frontal assessment battery score of control subjects at age 40–79 (N = 80; 40 men and 40 women; mean age, 59.0 ± 11.1 years)*.

* *p < 0.05*.

### Magnetic Resonance Imaging and Positron Emission Tomography Imaging

Before the PET scan, MRI (0.3 T MRP7000AD; Hitachi, Tokyo, Japan) was performed with 3-dimensional mode sampling (TR/TE: 200 ms/23 ms, 75° flip angle, 2 mm slice thickness with no gap, 256 × 256 matrices). MRI data were used to determine suitable areas for regions of interest (ROI) analysis. With the aid of data relating to tilt angle and spatial coordinates obtained during the procedure for determining the intercommisural (AC-PC) line on each subject's sagittal MR images, a PET gantry was set parallel to the AC-PC line by tilting and moving the gantry for each study. This enabled us to reconstruct the PET images parallel to the AC-PC line without reslicing (15).

The details of the PET procedure are described elsewhere (16). In brief, we used a high-resolution brain PET scanner with 24 detector rings yielding 47-slice images simultaneously (SHR12000; Hamamatsu Photonics KK, Hamamatsu, Japan). After head fixation using a thermoplastic facemask and a 10-min transmission scan for attenuation correction, serial scans (time frames: 4 × 30 s, 20 × 60 s, and 14 × 300 s) were performed for 92 min after slow bolus venous injection of a 6 MBq/kg dose of [^11^C]CFT. Three hours later, the [^11^C]FMZ PET study was started by conducting serial scans (time frames: 4 × 30 s, 20 × 60 s, and 8 × 300 s) for 62 min after slow bolus venous injection of a 6 MBq/kg dose of the tracer. No arterial blood sampling was performed in the present study. The chronological order of the two PET studies was counterbalanced among the groups.

### Image Data Processing

Polymorphic ROIs were located bilaterally on the caudate, putamen, cerebellum and VTA on the MR images (17). The ROIs were then automatically transferred onto the corresponding [^11^C]CFT distribution images (reconstructed from images taken 70 to 90 m after the tracer injection) using a commercially available image processing software (Dr. View, Asahi Kasei Co, Tokyo, Japan) that deals with DICOM data and generates parametric images of PET data, working on a SUN workstation (Hypersparc ss-20, SUN microsystems, San Diego, CA, USA) (16). We calculated the reference tissue-derived ratio index (SUVR) (i.e., the ratio of the PET count in the target region to the PET count in the cerebellum) in the late integrated image because the value of this ratio reflects the binding potential estimated by the quantitative 3-compartment 4 parameter model for [^11^C]CFT, which we tested previously (16). The PET image used for ROI setting was composed of two consecutive images that covered slices of 6.8 mm thickness in the z direction (18), so each ROI value contained functional information about the striatum to a depth of at least 6.8 mm in the z direction (volume data). In the [^11^C]FMZ study, the non-displaceable binding potential (BP_ND_) in each region was estimated using time-activity curves of target and reference (Pons) regions based on the simplified reference tissue method followed by the Logan plot method, in which k2^*^ derived from SRTM was used for the Logan analysis (19–23). The parametric maps of [^11^C]CFT SUVR and [^11^C]FMZ BP_ND_ were normalized to Montreal Neurologic Institute (MNI) standard brain using statistical parametric mapping software (SPM12; http://www.fil.ion.ucl.ac.uk/spm). After smoothing images with an isotropic Gaussian kernel of 6 mm FWHM, t statistics were performed on a voxel-by-voxel basis without gland mean scaling for contrasts of the between-group condition, resulting in t-statistic maps (SPM{t}). Subsequently, these maps were transformed to the unit normal distribution (SPM{Z}).

### Statistics

Prior to the voxelwise analyses, symptom-based hemispheric side differences in the PD group were effaced by flipping images so that the side of more affected striatal region contralateral to the more severe parkinsonian symptoms was set on the right in all PD images (Supplementary Figure 1). In SPM, group comparisons were performed using two-sample *t*-tests to examine the regional differences in the [^11^C]FMZ BP_ND_ between the PD group and the normal control group using age and sex as covariates of no interest. Voxelwise multiple regression analyses were performed to compare the levels of [^11^C]FMZ BP_ND_ with the clinical variables. The statistical threshold for significance was set to *p* < 0.001 and was uncorrected for multiple comparisons with no extent threshold. Regarding this threshold without multiple comparison, because the cognitive severity of patients examined in the present study was minimal, a smaller change in [^11^C]FMZ binding was expected and the current study was therefore exploratory for the GABAergic dysfunction in early PD. Thus, we considered the statistics without multiple comparison in this [^11^C]FMZ analysis acceptable.

In the same ROI analysis, direct correlation analyses were performed between [^11^C]FMZ BP_ND_ in the prefrontal cortex and [^11^C]CFT SUVR in regions of the striatum and ventral tegmental area (VTA). The p-value below the Bonferroni-corrected threshold (*p* < 0.05/6 = 0.0083) was considered to be significant.

## Results

### Demographic and Clinical Characteristics of the Study Participants

The participants' demographic and clinical characteristics are presented in Table 1. No significant differences in the sex ratios were observed between the patients with PD and controls. Twelve out of thirteen PD patients showed normal MMSE scores (≥24). The MMSE score of one patient was 19 because of the severe tremor that disrupted his hand maneuvers. The total FAB scores were significantly lower in the patients with PD than in age-matched healthy controls.

### Levels of CFT Binding

As reported previously (16), the levels of [^11^C]CFT SUVR were consistently found to be significantly lower in the bilateral putamen, caudate and VTA in patients with PD than in controls (Table 2). The [^11^C]CFT SUVR level on the more affected side was reduced more than that on the less affected side in the population with severe H&Y I-II outcomes. Supplementary SPM analysis with a two-sample *t*-test between the PD and control groups confirmed right-sided (affected side) dominance of the reduction of [^11^C]CFT SUVR (Supplementary Figure 1).

**Table 2:** Levels of [^11^C]CFT uptake ratio (SUVR).

**Region of interest (ROI)**	**Side**	**Parkinson's disease group (*N* = 13)**	**Control group (*N* = 6)**
Putamen	More affected (right)	2.26 ± 0.17^#^	3.99 ± 0.43
	Less affected (left)	2.59 ± 0.26^#^	4.05 ± 0.57
Caudate	More affected (right)	2.92 ± 0.34^#^	3.70 ± 0.42
	Less affected (left)	3.13 ± 0.35^#^	3.61 ± 0.30
VTA		1.59 ± 0.20^#^	1.84 ± 0.24

*Data are presented as the mean ± S.D*.

*Paired t-tests were used to compare data between two groups*.

# *p < 0.05 vs. control group*.

### Whole Brain Analysis: Comparison of [^11^C]FMZ BP_ND_ Between PD Patients and Controls

**Figure 1:**
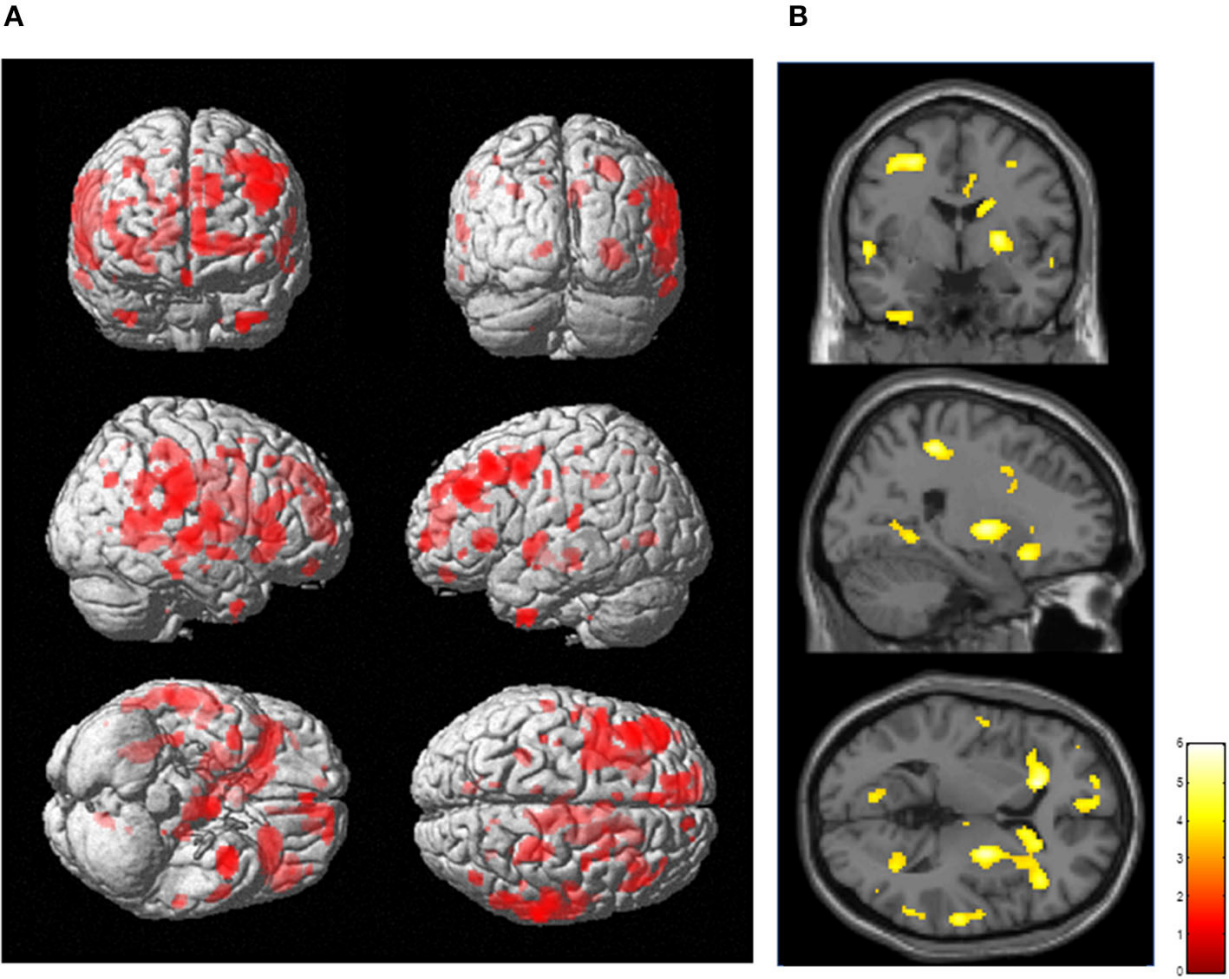
Reduced cerebral [^11^C]FMZ BP_ND_ in the Parkinson's disease group relative to the control group. The statistical threshold was set at *P* < 0.001, uncorrected multiple comparisons. **(A)** Results are shown on a three-dimensional rendering of the brain surface. Color bar represents the T value. The patient group showed significant cerebral reduction of [^11^C]FMZ BP_ND_ predominantly in the bilateral frontal and temporal cortex (shown in red). **(B)** Results are shown on canonical T1 images. The patient group showed a significant reduction in [^11^C]FMZ BP_ND_ on the more affected side of the basal ganglia (shown in yellow).

The results of SPM for [^11^C]FMZ BP_ND_ in the PD group compared with the control group showed significant reductions in [^11^C]FMZ BP_ND_ in the cortical areas, including the frontal, parietal, and temporal cortices, especially on the more affected side (right side) (Figure 1A; Table 3A). In addition, reduced [^11^C]FMZ BP_ND_ was also observed on the bilateral caudate and on the more affected side (right side) of the putamen (Figure 1B; Table 3A).

**Table 3:** Statistical parametric mapping (SPM12) results.

**Anatomical region**	**BA**	**Talairach coordinate**			**Cluster**	**Voxel**	**Z**	**Peak** ***P*****-value**		
		**x**	**y**	**z**	**size**	***T*-value**	**score**	**FWE**	**FDR**	**Uncorrected**
**(A) Regions of [**^**11**^**C]FMZ BP**_**ND**_ **reduction in patients with Parkinson's disease relative to controls**										
Left caudate		−17.8	25.3	0.6	282	5.99	4.64	*P* = 0.421	*P* = 0.375	*P* <0.0001
Left insula		−35.6	23.3	0.7		4.14	3.56	*P* = 1.000	*P* = 0.766	*P* <0.0001
Right superior temporal gyrus		61.4	21.3	1.1	409	5.79	4.53	*P* = 0.557	*P* = 0.375	*P* <0.0001
Right postcentral gyrus	43	53.5	−9.0	15.2		5.10	4.16	*P* = 0.957	*P* = 0.387	*P* <0.0001
Right parahippocampal gyrus	19	29.7	46.7	−1.0	348	5.76	4.52	*P* = 0.573	*P* = 0.375	*P* <0.0001
Right caudate		37.6	27.6	−8.7		3.82	3.34	*P* = 1.000	*P* = 0.814	*P* <0.0001
Right inferior parietal lobule	40	57.4	23.9	27.0	839	5.61	4.44	*P* = 0.681	*P* = 0.375	*P* <0.0001
Right superior temporal gyrus	22	59.4	40.2	11.2		4.29	3.66	*P* = 1.000	*P* = 0.724	*P* <0.0001
Right putamen		23.8	−5.6	4.0	1420	5.43	4.34	*P* = 0.805	*P* = 0.375	*P* <0.0001
Right inferior frontal gyrus	47	37.6	25.2	−1.3		5.16	4.19	*P* = 0.939	*P* = 0.375	*P* <0.0001
Left mammillary body		−2.0	−8.0	−4.6	304	5.35	4.30	*P* = 0.853	*P* = 0.375	*P* <0.0001
Left red nucleus		−5.9	−18.0	−10.9		4.43	3.75	*P* = 1.000	*P* = 0.678	*P* <0.0001
Right postcentral gyrus	3	25.7	−32.6	47.7	224	5.32	4.28	*P* = 0.868	*P* = 0.375	*P* <0.0001
Left precentral gyrus	9	−43.6	23.3	39.4	1298	5.30	4.27	*P* = 0.877	*P* = 0.375	*P* <0.0001
Left middle frontal gyrus	46	−43.6	36.3	25.8		5.23	4.23	*P* = 0.910	*P* = 0.375	*P* <0.0001
Right cingulate gyrus	31	9.9	−25.4	36.3	669	4.87	4.02	*P* = 0.992	*P* = 0.477	*P* <0.0001
**(B) Regions of [**^**11**^**C]FMZ BP**_**ND**_ **that were positively correlated with the FAB total score in patients with Parkinson's disease**										
Right anterior cingulate	24	4.0	29.0	−3.1	196	8.62	4.38	*P* = 0.969	*P* = 0.873	*P* <0.0001
Left anterior cingulate	24	−4.0	34.9	−1.7		5.51	3.56	*P* = 1.000	*P* = 0.873	*P* <0.0001
Left thalamus		−9.9	−31.2	−1.8	132	8.14	4.27	*P* = 0.990	*P* = 0.873	*P* <0.0001
Left putamen		−19.8	7.9	3.3	85	6.06	3.73	*P* = 1.000	*P* = 0.873	*P* <0.0001
Left claustrum		−35.6	0.4	7.4		5.03	3.38	*P* = 1.000	*P* = 0.873	*P* <0.0001
Left insula	13	−41.6	4.6	14.5	25	5.84	3.67	*P* = 1.000	*P* = 0.873	*P* <0.0001
Left superior temporal gyrus	22	−41.6	−51.5	19.2	19	5.60	3.59	*P* = 1.000	*P* = 0.873	*P* <0.0001
Left cuneus	17	−19.8	−79.1	11.3	13	5.34	3.50	*P* = 1.000	*P* = 0.873	*P* <0.0001
Left parahippocampal gyrus	36	−41.6	−22.4	−20.7	47	5.34	3.50	*P* = 1.000	*P* = 0.873	*P* <0.0001
Left inferior frontal gyrus	47	−37.6	28.9	−4.8	32	5.17	3.44	*P* = 1.000	*P* = 0.873	*P* <0.0001
Left middle frontal gyrus	10	−33.7	39.8	18.3	16	5.05	3.39	*P* = 1.000	*P* = 0.873	*P* <0.0001

*BA, Brodmann area; FWE, familywise error; FDR, false discovery rate; FAB, frontal assessment battery*.

### Whole Brain Analysis: Correlation Between [^11^C]FMZ BP_ND_ and PD-Related Clinical Factors

**Figure 2:**
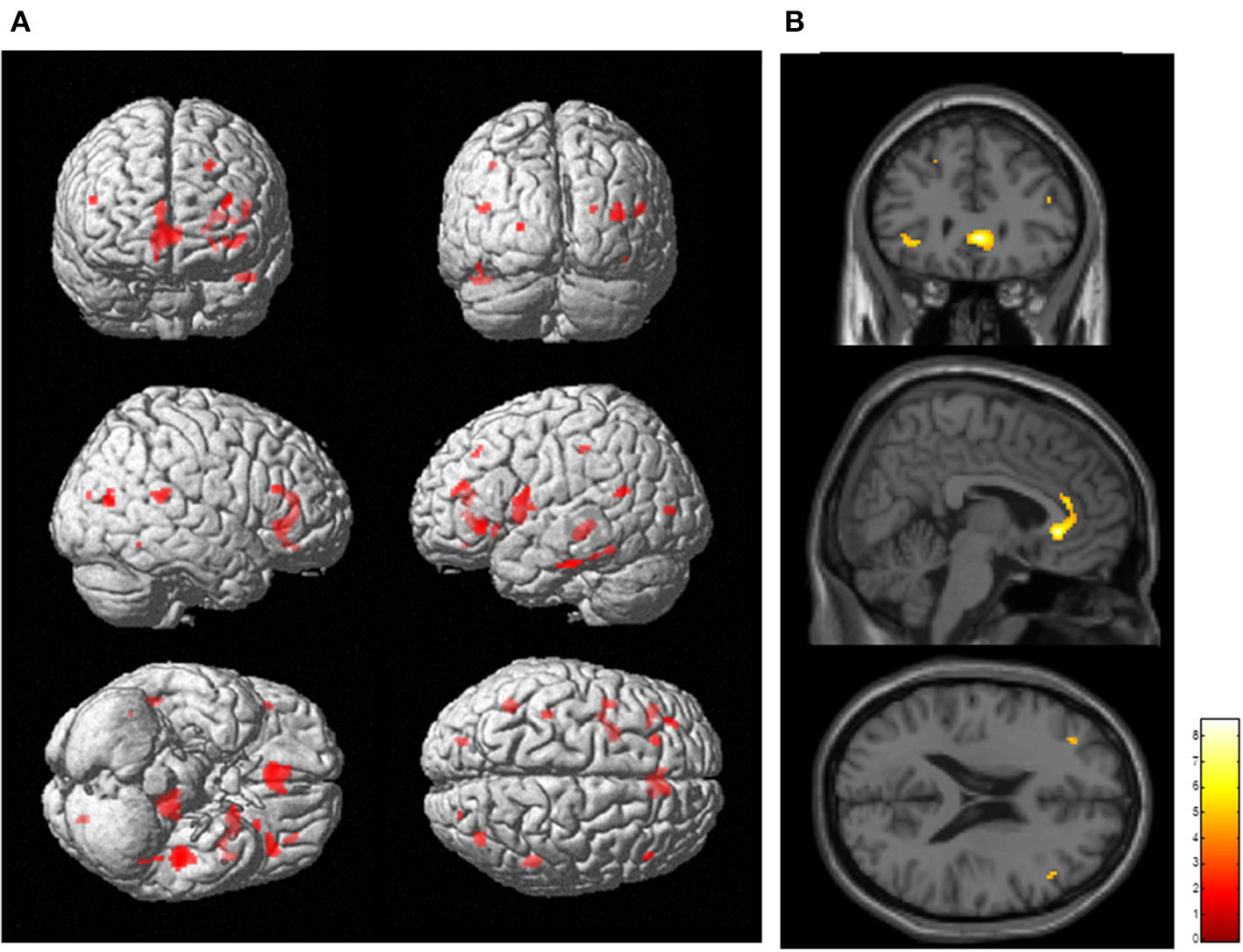
Correlation analysis between regional [^11^C]FMZ BP_ND_ and frontal assessment battery (FAB) total scores in patients with Parkinson's disease. The statistical threshold was set at *P* < 0.001, uncorrected multiple comparisons. **(A)** Results are shown on a three-dimensional rendering of the brain surface (shown in red). Color bar represents the T value. **(B)** The results are shown on canonical T1 images (shown in yellow). There was a significant positive correlation between the total FAB score and some frontal regions.

SPM analysis revealed that several clusters, especially in the frontal cortices and the left thalamus, were identified as the regions in which the total FAB score was positively correlated with the levels of [^11^C]FMZ BP_ND_ in the patients with PD (Figure 2; Table 3B). However, there was no significant correlation between MMSE or UPDRS part III and [^11^C]FMZ BP_ND_ in the patients with PD.

### ROI Analyses: The Relation Between [^11^C]FMZ BP_ND_ and [^11^C]CFT SUVR in the Same Striatal Regions in Patients in the PD Group

**Figure 3:**
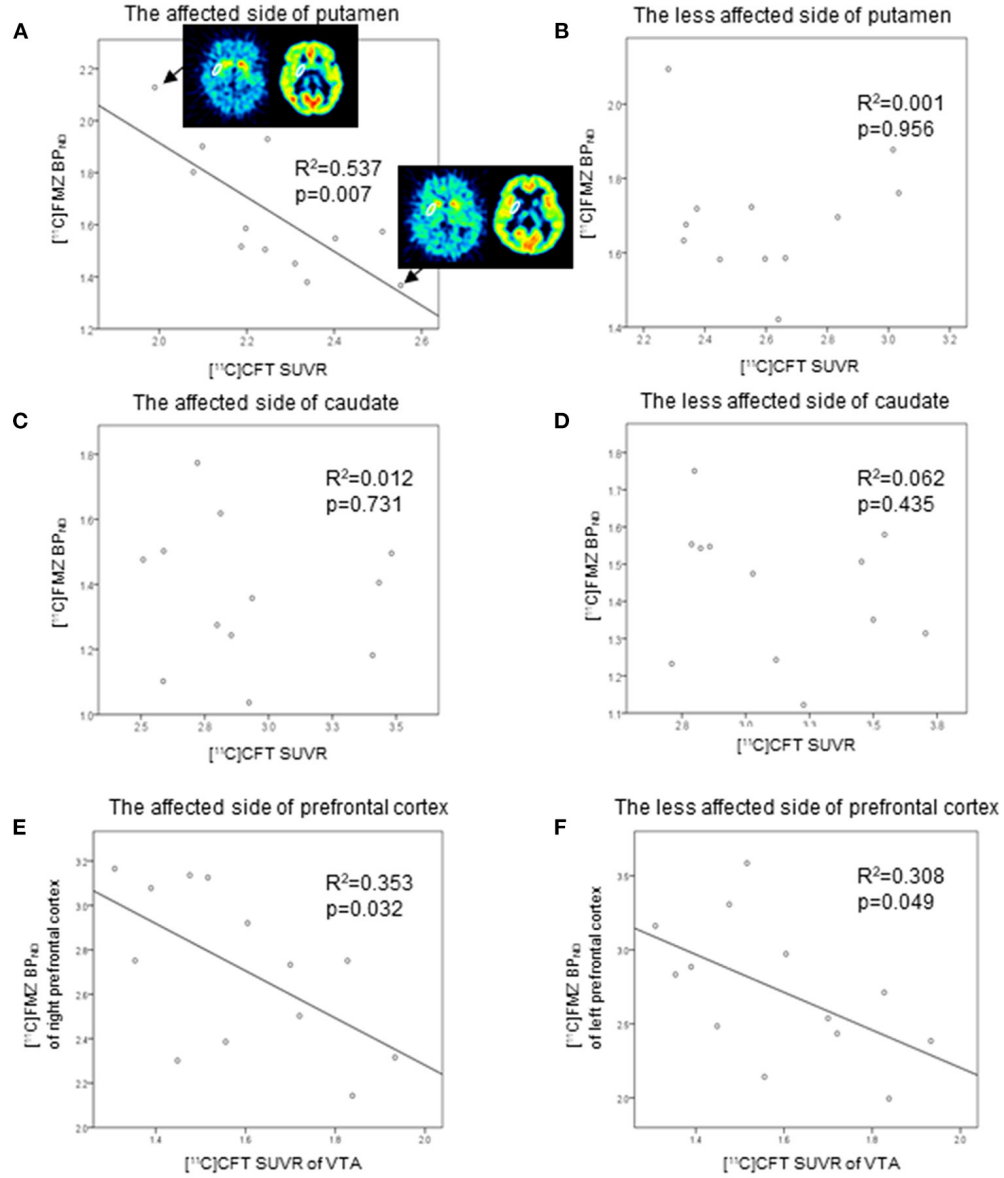
Scatter plot of the [^11^C]FMZ BP_ND_ and [^11^C]CFT standardized uptake value ratio (SUVR). The [^11^C]FMZ BP_ND_ was negatively correlated with [^11^C]CFT SUVR on the more affected side of the putamen **(A)** (corrected *p* < 0.05). The [^11^C]FMZ SUVR was not correlated with [^11^C]CFT SUVR **(B–D)** on the less affected side of the putamen or bilateral caudate. [^11^C]CFT SUVR in the VTA was tended to correlate negatively with [^11^C]FMZ BP_ND_ in the bilateral prefrontal cortical areas **(E,F)** (corrected *p* > 0.05).

Direct comparisons between [^11^C]FMZ BP_ND_ and [^11^C]CFT SUVR in the same ROIs showed significantly negative correlations on the affected side of the putamen in patients with PD (Figure 3A). However, there were no significant correlations between them on the less affected side of the putamen and the bilateral caudate (Figures 3B–D). There was a tendency toward negative correlations between [^11^C]CFT SUVR in the VTA and [^11^C]FMZ BP_ND_ in the bilateral prefrontal cortical areas (Figures 3E,F).

In the control group, there were significant positive correlations between them on one side (left) of the putamen. There was a tendency toward positive correlations between them when the results of the bilateral putamen were combined (Supplementary Figure 2).

## Discussion

In the present study, we showed a significant decrease in [^11^C]FMZ binding in the striatum and frontal to parietal-temporal cortex in PD patients at H&Y stage 1 or 2. Reduced FABs scores were significantly correlated with decreased [^11^C]FMZ mainly in the frontal area. On the affected side of the putamen, [^11^C]FMZ BP_ND_ was lower in PD patients than in the control group, and [^11^C]FMZ BP_ND_ was negatively correlated with [^11^C]CFT SUVR. These results might be the first to elucidate the *in vivo* mutual relation between dopamine and GABA system alterations in early PD, which has long been suggested in many animal experiments and clinical trials.

A rule of thumb in degenerative diseases such as PD is that the relations among neurotransmitter systems vary during disease progression. Actually, the pathological change of PD requires years to fully develop in the central nervous system and reaches the neocortex in its final stage (24). In this study, the fact that patients were at H&Y stage 1 or 2 indicates that the pathological change (chiefly α-synuclein) in the central nervous system was thought to reside in the brainstem, suggesting that mild abnormality of motor function is manifested clinically. Despite this, cortical [^11^C]FMZ binding was decreased even at this stage in the patient group compared with the control group. A recent study showed a disruption of functional brain networks within the whole brain even in the early stage of PD (25). The functional responses in the cerebral cortex depend on the degree of excitatory and inhibitory neurons working purposefully. It is well known that inhibitory interneurons are providers of the GABAergic system, which is often dysfunctional in several psychiatric disorders (26). Therefore, the present finding of reduced [^11^C]FMZ binding in the cerebral cortex in the PD group may indicate an impairment of GABA function in cortical interneurons, which may result from abnormalities in the nigrostriatal-cortical loops because cortical [^11^C]FMZ binding was much lower on the dopaminergically affected side. A previous report that a disturbance in inhibitory interneurons in schizophrenia was associated with cognitive decline (27) suggests that a decrease in [^11^C]FMZ binding in the cerebral cortex may be a premonition that cognitive decline associated with dementia will develop in the future.

The contention of cortical GABA-related cognitive decline may be true in regard to the results found in the cognitive function-PET parameter relation in the present study. In addition to motor impairments, PD patients display various cognitive impairments, ranging from mild cognitive impairments to dementia, during their clinical course. It is well known that frontal lobe dysfunction exists even at an early stage of PD (2). The FAB is a composite battery to evaluate executive function, and accordingly, the neuropsychological finding in the present study was in line with previous reports showing that frontal dysfunction dominates in early stages of PD (28). Furthermore, the frontal cortex was highlighted as the region in which [^11^C]FMZ binding was positively correlated with the total FAB score but not the MMSE score. Because our patients with PD at an early stage of severity are far from suffering dementia that will come at a late stage (1), it is no wonder that our patients had normal MMSE scores, which is used to assess general cognition (29). The normal range in MMSE scores might also be responsible for the lack of significant correlation on [^11^C]FMZ binding. A look at dopaminergic dysfunction in motor impairment will give us a general idea of the abnormalities in the nigrostriatal-cortical circuit, where the electrophysiological mechanism of cellular responses altered in PD is introduced (30). Hence, striatal dopamine transporter imaging is advantageous for determining the degree of impairment in dopamine neurons, which would correlate not only with the severity of motor symptoms in patients with PD (31) but also with cognitive deterioration during its clinical course (32). While there was no significant correlation between [^11^C]CFT binding and FAB score in the present study, since the major target of the nigro-striatal-cortical loop is the frontal cortex, it is conceivable that dopaminergic dysfunction would affect frontal activities to some extent. Indeed, striatal binding of ^123^I-ioflupane was reported to correlate with attention and executive function in PD with normal MMSE (33). Instead, our result that decreased [^11^C]FMZ binding in the frontal lobe is considered to reflect a disruption of neuronal integration governed partly by the GABAergic system.

In the present study, we showed correlations between [^11^C]CFT and [^11^C]FMZ binding within the striatum. According to the classical model of dopaminergic modulation in the nigrostriatal-cortical circuit, dopamine depletion causes greater inhibitory input in the striatum (direct pathway), resulting in an augmentation of the final inhibitory output to the thalamus and cerebral cortex (13). This evidence convinces us of an alteration of the balance in dopamine/GABA activity in the striatal region of patients with PD. Indeed, while the relation between [^11^C]CFT and [^11^C]FMZ binding in the putamen was found to be positively correlated in the healthy control group (Supplementary Figure 2), the result in the PD group showed a negative correlation in the affected putamen (Figure 3A). The negative correlation in the PD group might suggest that the more severe dopamine demise becomes, the smaller GABA transmission or a downregulation of GABA function (a larger capacity of [^11^C]FMZ uptake) occurs in the affected putamen. Due to the limited spatial resolution of our PET camera, the globus pallidus interna was beyond inspection, and the issue of the GABA/dopamine relation in this region remains to be explored. Judging from a different view, a decrease in [^11^C]FMZ binding in the putamen of PD patients in the present study might be ascribed to the reduction in GABA_A_ receptors expressed on nigrostriatal neurons. While experimentally excessive GABA suppresses dopamine release in an early PD animal model (14), it is likely that a persistent reduction in GABA transmission in the putamen might lead to downregulation of GABA_A_ receptors, resulting in a reduction in [^11^C]FMZ binding in our chronically ailing patients. Since there was a tendency of negative correlation between the VTA [^11^C]CFT binding and prefrontal [^11^C]FMZ binding, it is very likely that an abnormality of GABA/dopamine relation might exist in not only the nigrostriatal but also mesocortical dopaminergic systems. A lack of significance in the mesocortical system might be due to the fact that the dopaminergic dysfunction found in the VTA was milder than that in the putamen in early PD.

In the present study, there are a couple of limitations to disclose. The number of participants was small, which makes the current results preliminarily and needs further study to confirm them. We did not measure the exact concentration of GABA, which could instead be estimated by measuring the availability of GABA_A_ receptors indirectly with [^11^C]FMZ. In this respect, a simultaneous MRS measurement would have been preferable, but there is methodological difficulty with MRS in that the search region (volume of interest) is too large to exclude regions outside the striatum. We used polymorphic ROIs, but it might be more suitable to use atlas-based fixed VOI, which enables more consistent assessment between subjects.

In conclusion, this study showed that cortical GABAergic dysfunction exists even at an early stage of PD and is associated with frontal cognitive dysfunction. Although GABA dysfunction coexists with dopaminergic loss in the putamen, a negative correlation between them in PD suggests that further dopamine demise would generate downregulation of GABA function in the striatum (augmentation of inhibitory output to the cortex ultimately). Combined neurofunctional imaging and neuropsychological assessment could help elucidate the underlying neural mechanism and cognitive impairments in PD.

## Data Availability Statement

The original contributions presented in the study are included in the article/Supplementary Material, further inquiries can be directed to the corresponding author/s.

## Ethics Statement

The studies involving human participants were reviewed and approved by the Ethics Committee of Hamamatsu Medical Center. The patients/participants provided their written informed consent to participate in this study.

## Author Contributions

HT, TT, and YO contributed to conception and design of the study. All authors contributed to manuscript revision, read, and approved the submitted version.

## Funding

This work was supported by JSPS KAKENHI Grant Number: JP16H06402.

## Conflict of Interest

The authors declare that the research was conducted in the absence of any commercial or financial relationships that could be construed as a potential conflict of interest.

## Publisher's Note

All claims expressed in this article are solely those of the authors and do not necessarily represent those of their affiliated organizations, or those of the publisher, the editors and the reviewers. Any product that may be evaluated in this article, or claim that may be made by its manufacturer, is not guaranteed or endorsed by the publisher.
