# Supplementary material for: In vivo Illustration of Altered Dopaminergic and GABAergic Systems in Early Parkinson's Disease
Source: Front Neurol. 2022 May 17;13:880407. doi: 10.3389/fneur.2022.880407 (PMC9152017; doi:10.3389/fneur.2022.880407)
Supplement: Supplementary file 1 [file Table_1.DOCX]

Supplementary Material

# Supplementary Data

**Supplementary Figure 1.** SPM analysis of the between-group comparison of [^11^C]CFT SUVR. Significant reduction of [^11^C]CFT SUVR in the affected side of the putamen (p<0.05, familywise error correction).

**Supplementary Figure 2.** Scatter plot of the [^11^C]FMZ BP_ND_ and [^11^C]CFT standardized uptake value ratio (SUVR) in the control group. The [^11^C]FMZ BP_ND_ was positively correlated with [^11^C]CFT SUVR on one side (right) of the putamen (A). There was a tendency toward a positive correlation between them in the bilateral putamen.
