# Supplementary figures and images for: In vivo Illustration of Altered Dopaminergic and GABAergic Systems in Early Parkinson's Disease
Source: Front Neurol. 2022 May 17;13:880407. doi: 10.3389/fneur.2022.880407 (PMC9152017; doi:10.3389/fneur.2022.880407)

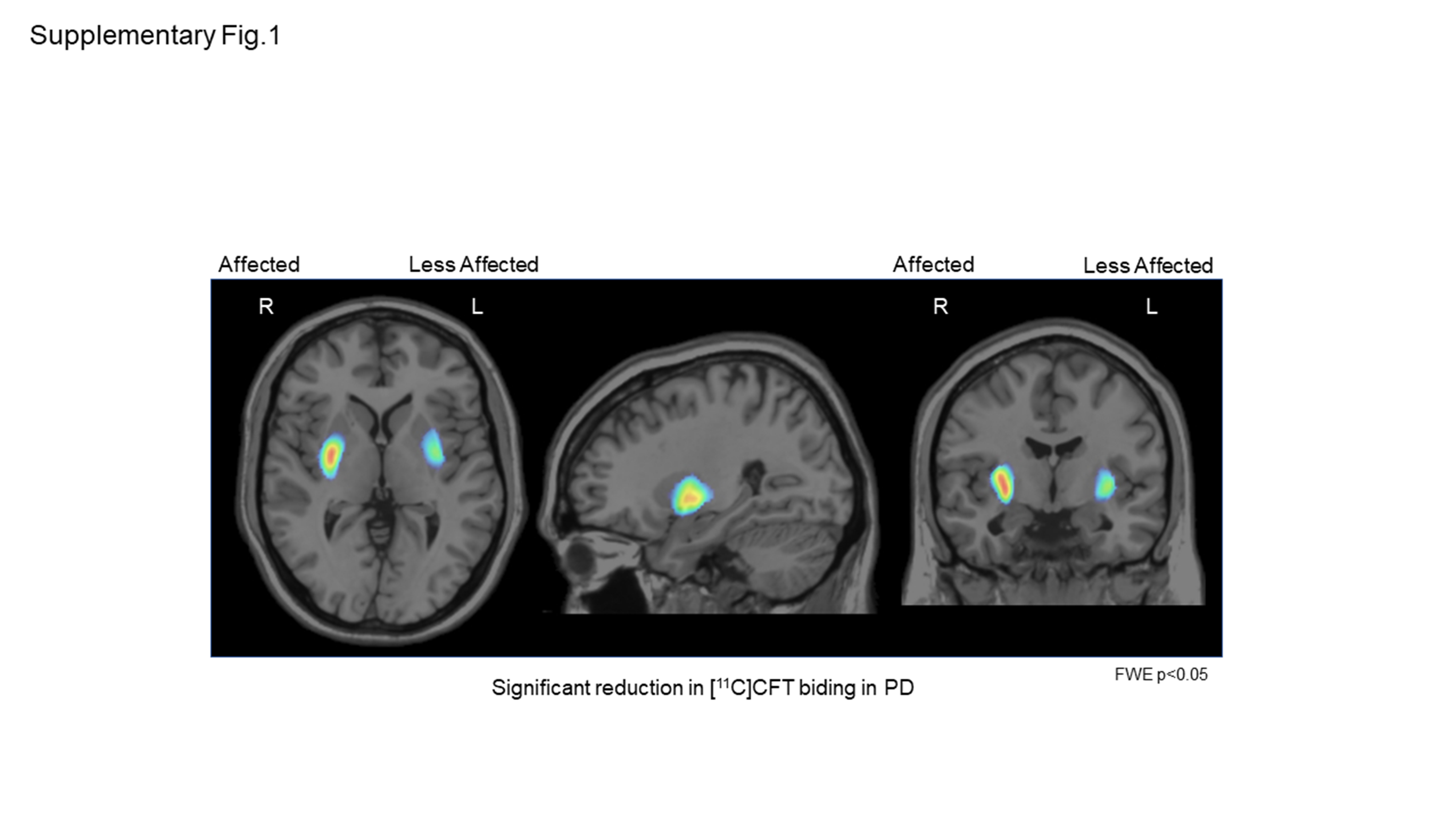

Supplement: Supplementary file 2 [file Image_1.TIF]

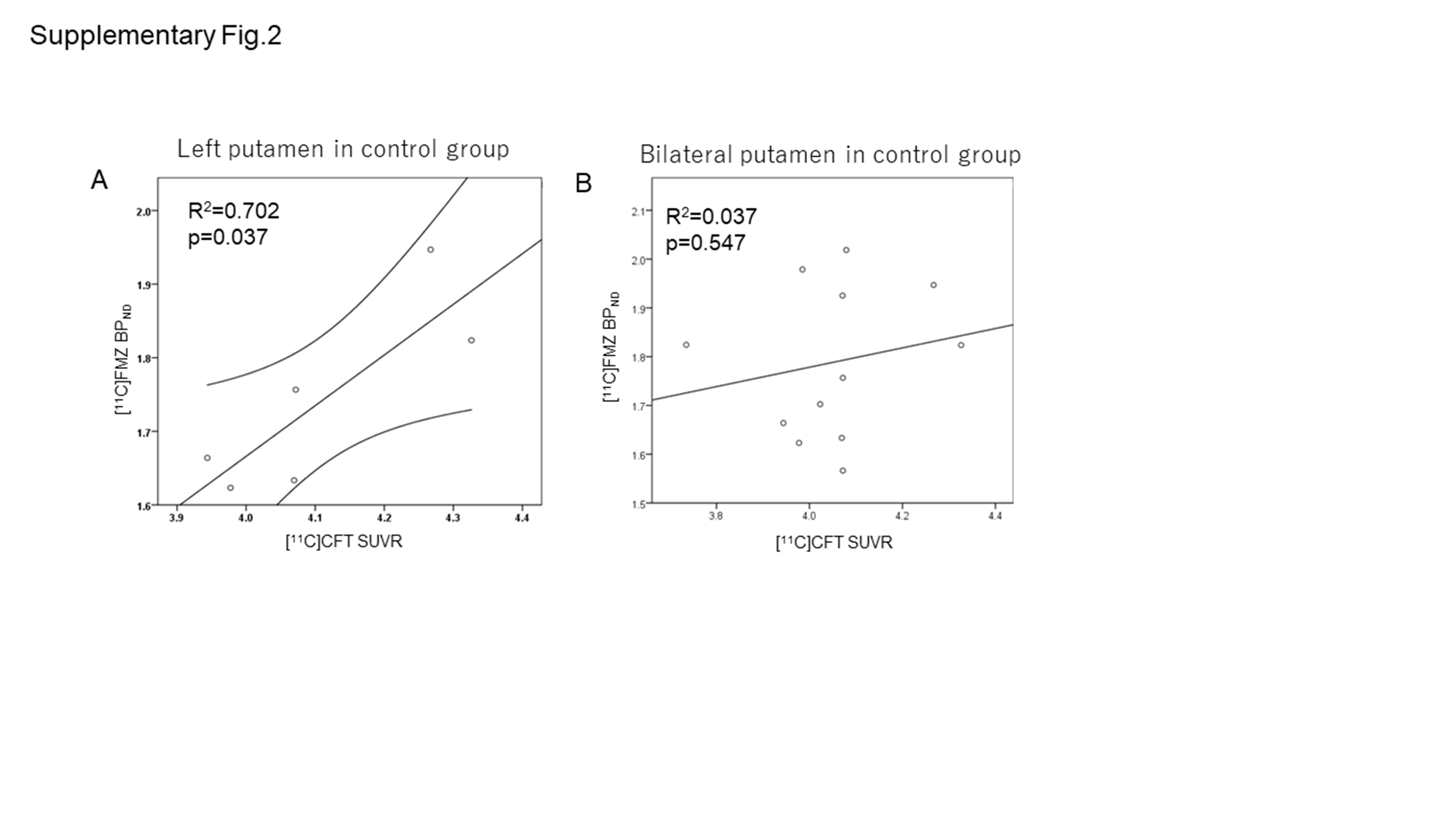

Supplement: Supplementary file 3 [file Image_2.TIF]
